# BLOOD MULTIOMIC PROFILES REFLECT SYSTEM STATES OF ORGANS IN MICE

**DOI:** 10.1093/geroni/igae098.3637

**Published:** 2024-12-31

**Authors:** Kengo Watanabe, Lance Pflieger, Max Robinson, Jodi Lapidus, Richard Miller, Oliver Fiehn, Robert Moritz, Noa Rappaport

**Affiliations:** Institute for Systems Biology, Seattle, Washington, United States; Phenome Health, Seattle, Washington, United States; ISB, Seattle, Washington, United States; OHSU-PSU School of Public Health, Portland, Oregon, United States; University of Michigan, Ann Arbor, Michigan, United States; UC Davis, Davis, California, United States; Institute For Systems Biology, Seattle, Washington, United States; Institute for Systems Biology, Seattle, Washington, United States

## Abstract

The rate of aging can vary among organs, as evidenced by organ-specific biological age models. Such models are typically constructed based on the blood biomarkers or phenotypes that are known to be specific to a particular organ. However, this approach potentially limits the models to reflecting only a narrow scope of organ system states. Herein, we report the potential of blood omics data to reflect alterations in organ systems, using the NIA Longevity Consortium mouse prolongenvity proteomics and metabolomics data which were derived from kidney, liver, gastrocnemius muscle, and plasma samples from each mouse. While correlation analysis mainly identified the correlations of matched analytes between organs and blood, machine learning models to predict the organ analyte abundance or system state from the plasma analytes revealed the differences between sexes or prolongevity interventions. Our findings suggest the power of blood omics for identifying and characterizing diverse system states of organs involved in aging and longevity.

